# Political Prioritization of Access to Medicines and Right to Health: Need for an Effective Global Health Governance Through Global Health Diplomacy: Comment on "More Pain, More Gain! The Delivery of COVID-19 Vaccines and the Pharmaceutical Industry’s Role in Widening the Access Gap"

**DOI:** 10.34172/ijhpm.8578

**Published:** 2024-08-19

**Authors:** Vijay Kumar Chattu, Anjali Pushkaran, Prakash Narayanan

**Affiliations:** ^1^Centre for Global Health Research, Saveetha Medical Collage and Hospital, Saveetha Institute of Technical Sciences, Saveetha University, Chennai, India; ^2^Temerty Department of Medical Sciences, University of Toronto, Toronto, ON, Canada; ^3^Department of Community Medicine, Faculty of medicine, Datta Meghe Institute of Medical Sciences, Wardha, India; ^4^Department of Health Policy, London School of Economics and Political Science, London, UK; ^5^Center for Evidence-based Diplomacy, Global Health Research and Innovations Canada Inc. (GHRIC), Toronto, ON, Canada.; ^6^Department of Health Policy, Manipal Academy of Higher Education, Manipal, India.

**Keywords:** Global Health, Governance, Diplomacy, Access to Medicines, Political Prioritization, Equity, COVAX, COVID-19

## Abstract

Borges and colleagues’ article entitled "More Pain, More Gain! The Delivery of COVID-19 Vaccines and the Pharmaceutical Industry’s Role in Widening the Access Gap," analyzes the role of pharmaceutical companies in providing equitable access to COVID-19 vaccines. They concluded that with the failure of COVID-19 Vaccine Global Access (COVAX), the health gaps have widened due to the profit-driven pharmaceutical sector. In this commentary, we highlight the role of COVAX and its attempt to bridge some access gaps since its inception and the need for reforms in policy-making and global health governance. The commentary highlights the role of global health diplomacy in promoting equity and negotiating the Trade-Related Aspects of Intellectual Property Rights (TRIPS) waiver for COVID-19 vaccines at the World Trade Organization (WTO) thereby promoting global solidarity, global partnerships, access to medicine and health products, and the right to health. We conclude that political prioritization is the key to balance the impact of profit-driven pharma industry and addressing the needs of low- and middle-income countries (LMICs).

## Background

 Borges and colleagues in their article “More Pain, More Gain! The Delivery of COVID-19 Vaccines and the Pharmaceutical Industry’s Role in Widening the Access Gap” reported the negative impacts of profit-driven pharmaceutical sector by widening the access gaps between developed and developing nations.^1^ The authors made an excellent effort to analyze the access to COVID-19 vaccines and rightly highlighted the pharmaceutical industry’s maximalist behavior and attributed it to advance purchase agreements (APAs) with high-income countries (HICs). APAs are contractual agreements in which a buyer commits to purchasing a specified amount of a product from a supplier in advance of its production or availability. However, the COVID-19 pandemic has divided the world with a lack of global solidarity, strong global leadership, and international cooperation with the rise of vaccine nationalism thereby compromising the spirit of multilateralism and global partnerships. This scenario makes a good case for the need for an effective global health governance for medicines and vaccine supply and the world has witnessed the importance and rise of global health diplomacy between nations and at various regional and global platforms.

## COVAX and Vaccine Nationalism

 We like to bring to the attention that COVID-19 Vaccine Global Access (COVAX), the largest vaccine supply operation in history, has also used almost similar mechanism to Advance Market Commitment (AMC) for vaccines to fund the low- and middle-income countries (LMICs) that cannot afford to pay for COVID-19 vaccines. AMCs are financial agreements designed to stimulate the development and production of new products, particularly vaccines and other critical health interventions, by guaranteeing a future market for these products. AMCs are typically used to incentivize pharmaceutical companies to invest in research and development for diseases that predominantly affect low-income countries, where the potential for high returns on investment might otherwise be limited. COVAX made multiple AMCs with pharma companies but was pushed to the back of the queue as the manufacturers preferred delivery to HICs.^2^ The more pressing problem is the lack of transparency of pharma company contracts, their order books, and delivery times that they deliberately delayed vaccine delivery to COVAX. The COVAX agreements with the pharma companies were also not transparent since CEPI and GAVI have not made any of this public. So, assigning APAs as the primary factor responsible for COVAX failure is a highly stretched argument. The transparency in deals with pharma companies can contribute to more equitable differential pricing. A perfect example is the lessons learned from South Africa’s vaccine purchase deals. The pharma giant Johnson and Johnson charged South Africa $10 a dose, 15% more than the company charged the European Union, and the government was required to pay a non-refundable down payment of $27.5 million. This happened because the vaccine deals were not transparent and pharma companies could charge any price they want. Besides a recent scoping review on COVAX’s performance after three years also calls for reforms in global health governance and policy-making for the effective functioning of COVAX.^2^ This included filling holes of COVAX public-private partnership framework such as allowing HICs to strike bilateral deal outside COVAX, inadequacy of World Health Organization (WHO) allocation mechanisms to contain the pandemic, etc.^2^

 It is indisputable that vaccine nationalism caused significant equity gaps in COVID-19 vaccine access and was one of the major implementation challenges of COVAX.^1,3^ The paper views pharma companies’ profiteering approach as the prime reason that hinders global equitable access to medicines. However, it is important to acknowledge the remarkable achievement of the pharma industry that the COVID-19 vaccines were developed in less than 100 days.^4^ Most of the COVID-19 vaccine Research and Development indeed received public funding from HICs and they prioritized delivery to these countries. The APAs signed by pharma companies depict only one side of the equity problem as HIC governments promoted the nationalist strategy for their own economic and political gain. The governments as buyers are equally responsible for widening the gap of COVID-19 vaccines between Global North and South. COVAX faced a lack of funding and participation of HICs. Due to the preference for nationalist aspirations of the governments, more funding was allocated to independent national programs rather than COVAX. For example, Operation Warp Speed by the United States is a public-private partnership model for vaccine development with an initial budget of US$1 0 billion.^5^ Three dozen countries bypassed COVAX and made huge bilateral deals with manufacturers. It is this nexus of government and pharmaceutical companies that jeopardized the global COVID-19 vaccine equity.^2^

 Furthermore, the COVAX framework has several gaps that contributed to its failure. The alliance allowed the members to engage in bilateral deals outside of COVAX. It allowed the HIC members to procure doses up to 50% of their population through the facility through the “Optional Purchase Agreement,” whereas, for aided countries, the limit is 20%. An optional purchase agreement under COVAX allows participating countries to secure the option to purchase additional vaccine doses beyond their initial allocations, providing flexibility to meet varying vaccination needs. This mechanism ensures that countries can access more vaccines if required, while still benefiting from the pooled procurement and negotiated pricing of the COVAX initiative.^2^ This prima facie violates the principle of equity to which the alliance dedicates itself. This also contributed to fewer vaccines to LMIC and low-income countries via COVAX because COVAX was under a contractual obligation to give vaccines to HICs.

## Intellectual Property Rights and Global Health Diplomacy

 The pharma industry lobbied the World Trade Organization (WTO) to reject the intellectual property rights (IPR) waiver put forward by India and South Africa and later adopted a diluted version of the original proposal.^6,7^ This shows how tough it is to form a political consensus on the subject of IPR. The Trade-Related Aspects of Intellectual Property Rights (TRIPS) agreement has the provision of compulsory licensing by governments but was seldom used to produce COVID-19 vaccines because of the complexity of m-RNA technologies and lack of transfer of the same. WHO established the COVID-19 technology access pool in 2020, and not even a single pharma company shared the technology till 2022.^2^ To balance private rights and the right to health means to balance the incentive to innovation vs access to medicines. This is only achievable if there is a political consensus between all stake holders and establish a global health governance mechanism through diplomacy. But this is a complex process as depicted by the ongoing Pandemic Treaty negotiations where there are disagreements on sharing of data and intellectual property. Global health diplomacy which involves negotiations among multiple stakeholders for developing global policies has a potential role in addressing the challenges and filling the gaps created by the inaccessibility to essential medicines and drugs.^8^ Various authors have emphasized the critical role of global health diplomacy for addressing inequities, improving trade and development, strengthening international cooperation and global health security through effective global health governance.^9,10^

 It is not feasible to strip the pharma companies’ rights of IPR which is the core of production of new vaccines and medicines. What we need is a pandemic-specific governance treaty as pharma companies are integral parts of all the multi-lateral initiatives in global health. In the case of COVAX, its structure and mechanism of COVAX did not challenge the IPR. COVAX used AMC to bypass the IPR and the alliance was a middle-ground strategy between vaccine equity and IPR.^2^ The COVAX tried to accommodate the concepts of equity, global health as a public good and right to health and the structure and framework of the COVAX. Also, it is the responsibility of each government to protect its own citizens which caused the bilateral purchase spree outside the COVAX. These conflicts in interest were one of the major factors behind undermining of the COVAX. COVAX merely negotiated with pharma companies for lower price vaccines and did not enforce any capping for bilateral deals for its HIC participants.^2^ It is rightly pointed out the need for a TRIPS waiver or suspension for pandemic goods to allocate and distribute them more equitably.^3^ However, this waiver is helpful only if there is enough manufacturing capacity in other parts of the world to compete with the global north. If so, by theory more producers enter the vaccine market and subsequent price reductions must happen. The patenting system is so complex that the pharma companies can patent the vaccines, the manufacturing process, and the technology. Without the transfer of the technical know-how, the waiver of the patent would be of little use.

## Governance of Pharma Industry

 The writers argue for the strengthening of production capacity in LMICs to reduce the dependency on foreign manufacturers. It is definitely needed, and we think this suggestion ultimately boils down to the need for regulating pharma companies during a pandemic. Let’s assume that there are enough pharma companies with sufficient production capabilities in LMIC. The inherent motive of a pharma company is to make profits and there is no guarantee that these companies in LMICs will act on the notion of equity and vaccine accessibility to all. So, there is an undebatable need to have a complementary governance structure where the pharma companies could be regulated during a pandemic. However, alternatives such as public sector pharma manufacturing, open source drug development and university based drug development could also be helpful to manage the lack of manufacturing companies in LMICs.

 As pointed out by Borges and colleagues, during pandemic situations, the governments in LMICs may be able to strengthen their cooperation for control and management of the situation. But joint or shared procurement of essential medicines and high-demand technologies could be challenging because of low purchasing power, authority issues such as administrative and decision-making problems within the governments, geopolitics, poor infrastructure and technical capacities. COVAX supported the LMICs not only in procuring the medicines and vaccines but also helped them to secure required infrastructure and logistical arrangements (for example HOPE consortium to address logistics, shipping, and infrastructure needs). Also, the pooled procurement facilities from the United Nations International Children’s Emergency Fund and the Pan American Health Organization Revolving Fund to the Global Drug Facility helped to address some of these issues promptly. COVAX as a new organization had financial constraints and many countries that pledged financial support were slow to contribute financially. COVAX supported the LMICs not only in procuring the medicines and vaccines but also helped them to secure required infrastructure and logistical arrangements (for example HOPE consortium to address logistics, shipping, and infrastructure needs).

###  Price Regulations

 The exorbitant prices charged by the pharma companies affected the access to COVID-19 vaccines to LMICs. For example, Moderna sought to charge $42 per vaccine dose, four times the Pfizer offer, to the South African government (which never bought Moderna vaccine doses). Even at the Pfizer offer price, it would still be more highly priced than in Europe.^12^

 From the WHO data of market information on vaccine prices on Pfizer and Moderna vaccines, this is very evident.^11^

 The prices charged by Moderna and Pfizer vary hugely across the countries based on income classification. The HICs were charged more than double the prices of LICs and this is where the phenomenon of pandemic profiteering happened. The Figure 1 below depicts the profit made from vaccine sales by Moderna and Pfizer. In 2021, the profits were double for Pfizer, and for Moderna, from a loss of 0.34 billion, it made a profit of 12 billion just by vaccine deals (Table 1).^12,13^

**Table 1:** Prices of Pfizer and Moderna Vaccines in Different Countries

**Vaccine**	**LIC (Price in USD)**	**LMIC (Price in USD)**	**UMIC (Price in USD)**	**HIC (Price in USD)**
Moderna - mRNA-1273	10	10	28.8	25.5
Pfizer BioNTech - Comirnaty	7	10	12.5	20.67

Abbreviations: LIC, low-income country; LMIC, low- and middle-income country; UMIC, upper middle-income country; HIC, High-income country. Source: Market Information for Access to Vaccines (MI4A) – WHO.

 Due to the profits involved, the pharma companies delivered vaccines to COVAX (which earns less profits for them as the deal was for subsidized prices) very late. If there was a pricing policy for the pandemic goods, the pharma companies were less likely to discriminate between buyers. The COVID-19 vaccine market was complex amid the pandemic because of huge demand and proportionally driven prices. The high prices can be attributed to high demand and the pandemic-specific timeline (3-4 years to control the pandemic) does not have enough time for normal market competition to reduce the prices of the vaccines.

 Therefore, we need specific pricing mechanisms for the pandemic goods to stop the irrational pricing of vaccines by pharma companies. It is feasible to consider a price cap for vaccines across all countries to ensure no country pays more than the capped price than others and it stops the irrational pricing by pharma companies. Another option is to have a cost reimbursement policy for pandemic goods such as vaccines and therapeutics which is absent in many countries as of now. Through global health diplomacy, discussion among stakeholders and negotiation for efficient pricing mechanisms are possible which can ensure transparency and accountability. This will help to curb the monopoly pricings on pandemic vaccines. Dawes et al highlight that to truly create a more equitable future, for all population groups globally, leaders in health equity must possess a comprehensive understanding of the political determinants of health that influence health, in addition to their knowledge of social, environmental, behavioral and healthcare determinants.^14^

## Political declarations for “Right to Health” and “Access to Medicines”

 COVAX’s slogan is “No one is safe until everyone is safe,” which reflects a vital public health concern and the need for global cooperation. COVAX had the right motto but failed to deliver promised vaccines due to multiple framework gaps and implementation challenges. The COVAX alliance was built on the traditional model of aid financing for LMICs. The paper advocates for the right to health and its realization by the public to hold the stakeholders in multi-lateral alliances accountable. We argue for a “Whole of the Society” model where civil society, business partners, and government should be involved and collaborate in a way they are accountable to the public. It embodies a more inclusive strategy that goes beyond governmental bodies, involving a wide range of stakeholders such as individuals, families, communities, intergovernmental organizations, religious institutions, civil society, academia, the media, voluntary associations, and the private sector and industry.^15^ We can have successful negotiations implemented through global health diplomacy and ensure periodic assessments (monitoring and evaluation mechanisms) so that these policies get the feedback from all the stakeholders involved and make them accountable.

 Chattu et al have highlighted the role of global health diplomacy related to various political declarations and roadmaps for access to medicines (Table 2) argue for “Health as a human right” and emphasize multilateral collaborations and partnerships to address the equity gaps.^8^ However, working towards an international pandemic treaty where there are defined roles and responsibilities of the private health sector during pandemics across countries is very essential at the moment.^16^

**Table 2:** Summary of Political Declarations and Roadmap for Access to Medicines From 2000-2022^a^

**Global Organization/ Authority**	**Resolution/Year**	**Topic/Area**
OHCHR Commissioner	Report of the High Commissioner on COVID-19 vaccines (2022), A/HRC/49/35	The human rights implications of the lack of affordable, timely, equitable, and universal access and distribution of COVID-19 vaccines and the deepening inequalities between States highlight that vaccine delays have grave health consequences and other profound human rights implications
WHO	The 71^st^ World Health Assembly considered a report by the Director General (document A71/12), May 2018	Addressing the global shortage of and access to medicines and vaccines - road map for access to medicines, vaccines, and other related health products 2019–2023
OHCHR Report	A/HRC/47/23, May 2021	The central role of the State in responding to pandemics and other health emergencies, and the socioeconomic consequences thereof, in advancing sustainable development and the realization of all human rights
OHCHR Report	A/HRC/46/19, March 2021	Impact of the COVID-19 pandemic on the enjoyment of human rights around the world, including good practices and areas of concern
HRC	Human Rights Council resolution 46/14 of March 29, 2021	Ensuring equitable, affordable, timely, and universal access for all countries to vaccines in response to the COVID-19 pandemic
HRC	Human Rights Council resolution 41/10 of July 19, 2019	Access to medicines and vaccines in the context of the right of everyone to the enjoyment of the highest attainable standard of physical and mental health
UNGA	General Assembly resolution 73/3 of October 10, 2018	Political declaration of the high-level meeting of the General Assembly on the fight against tuberculosis
UNGA	General Assembly resolution 73/2 of October 10, 2018	Political declaration of the third high-level meeting of the General Assembly on the prevention and control of non-communicable diseases
UNGA	General Assembly resolution 71/3 of October 5, 2016	Political declaration of the high-level meeting of the General Assembly on antimicrobial resistance
UN Secretary-General	United Nations Secretary-General’s High-level Panel of September 2016	Access to Medicines (http://www.unsgaccessmeds.org/reports-documents) and the Panel’s report on promoting innovation and access to health technologies
HRC	Human Rights Council resolution 32/15 of July 1, 2016	Access to medicines in the context of the right of everyone to the enjoyment of the highest attainable standard of physical and mental health
UNGA	General Assembly resolution 70/1 of September 25, 2015	Transforming our world: the 2030 Agenda for Sustainable Development
HRC Social Forum	A/HRC/29/44 of 2015	Report of the 2015 Social Forum on Access to Medicines
HRC Special Rapporteur	A/HRC/23/42, May 1, 2013	Right of everyone to the enjoyment of the highest attainable standard of physical and mental health, Anand Grover, on access to medicines
HRC Special Rapporteur	A/63/263, August 11, 2008	The right to everyone to the enjoyment of the highest attainable standard of physical and mental health, Paul Hunt, on Human Rights Guidelines for Pharmaceutical Companies in relation to Access to Medicines
CESCR	Committee on Economic, Social and Cultural Rights, General comments No. 17 (2006)	States parties thus have to prevent unreasonably high costs for access to essential medicines, plant seeds, or other means of food production from undermining the rights of large population segments to health, food, and education
CESCR	Committee on Economic, Social and Cultural Rights, General comments No. 14 (2000)	Health facilities, goods, and services have to be accessible to everyone without discrimination within the jurisdiction of the State party. Accessibility has four overlapping dimensions: Non-discrimination, physical accessibility, economic accessibility, and information accessibility

Abbreviations: OHCHR, Commissioner of Office of the High Commissioner for Human Rights; WHO, World Health Organization; UNGA, United Nations General Assembly; HRC, Human Rights Council; CESCR, Committee on Economic, Social and Cultural Rights; UN, United Nations. ^a^ Reprinted from Chattu et al^8^ under the Creative Commons Attribution License (http://creativecommons.org/licenses/by/4.0/).

## Conclusions

 COVAX is structured based on a framework that emphasizes the collaboration between governments and corporations as the most effective approach to address market failures and recognize IPR as a catalyst for innovation. However, the pandemic profiteering by pharma companies, exorbitant pricing strategies, and rampant vaccine nationalism by the HIC governments weakened the alliance and jeopardized the global COVID-19 vaccine equity. The United Nations and WHO framework of ‘Right to health’ and notions of Global vaccine equity have to go hand in hand with incentives for innovation for pharma companies. We have seen the politics of IPR waiver in WTO and the current geopolitics makes it hard to construct an international treaty for pandemic goods. Future advocacy efforts could be aimed at convincing pharmaceutical companies to adopt a humanistic approach and help nations to achieve the “Right to health” and to convince or consider such requests from intergovernmental agencies during global public health emergencies.

## Ethical issues

 Not applicable.

## Competing interests

 Vijay Kumar Chattu is the Founder and CEO of Global Health Research and Innovations Canada Inc. (GHRIC) and leads the Center for Evidence-based Diplomacy. The other authors declare that they have no competing interests.
